# Endophytic *Pseudomonas fluorescens* promotes changes in the phenotype and secondary metabolite profile of *Houttuynia cordata* Thunb.

**DOI:** 10.1038/s41598-024-52070-y

**Published:** 2024-01-19

**Authors:** Kaifeng Wang, Zhannan Yang, Shiqiong Luo, Wenxuan Quan

**Affiliations:** 1 Key Laboratory for Information System of Mountainous Areas and Protection of Ecological Environment of Guizhou Province, Guizhou Normal University, Guiyang, Guizhou China; 2 School of Life Science, Guizhou Normal University, Guiyang, Guizhou China

**Keywords:** Biochemistry, Analytical chemistry, Bioanalytical chemistry, Mass spectrometry

## Abstract

The interactions between microbes and plants are governed by complex chemical signals, which can forcefully affect plant growth and development. Here, to understand how microbes influence *Houttuynia cordata* Thunb. plant growth and its secondary metabolite through chemical signals, we established the interaction between single bacteria and a plant. We inoculated *H. cordata* seedlings with bacteria isolated from their roots. The results showed that the total fresh weight, the total dry weight, and the number of lateral roots per seedling in the *P. fluorescens*-inoculated seedlings were 174%, 172% and 227% higher than in the control seedlings. *Pseudomonas fluorescens* had a significant promotional effect of the volatile contents compared to control, with *β*-myrcene increasing by 192%, 2-undecanone by 203%, decanol by 304%, *β*-caryophyllene by 197%, *α*-pinene by 281%, bornyl acetate by 157%, *γ*-terpinene by 239% and 3-tetradecane by 328% in *P. fluorescens*-inoculated *H. cordata* seedlings. the contents of chlorogenic acid, rutin, quercitin, and afzelin were 284%, 154%, 137%, and 213% higher than in control seedlings, respectively. Our study provided basic data to assess the linkages between endophytic bacteria, plant phenotype and metabolites of *H. cordata* to provide an insight into *P. fluorescens* use as biological fertilizer, promoting the synthesis of medicinal plant compounds*.*

## Introduction

Complex interactions between microbes and plants are established with the aid of signals from the plant host and environment^1–3^. These interactions are directly and/or indirectly affected by both pathogenic and beneficial bacteria^4–6^, and the complex chemical signaling also affects plant growth and development^4,5, 7^. Plants contain a large number of bacteria, some of which are beneficial, and can influence the phenotype and secondary metabolite profile of their plant hosts^8–10^, but there is a need to better understand these effects of the bacteria in order to possibly exploit such effects to greatly improve the productivity of the host plant^11^. However, few experimental or theoretical studies have investigated the influence of bacteria on the plant phenotype, which is associated with changes in the plant secondary metabolite profiles^12–15^. It have been reported that simplified, yet representative, synthetic bacterial model community consisting of seven species to inhibit *F. verticillioides* to directly or indirectly promote maize growth^16^, whereas colonization with *Streptomyces* sp. can promote host plant health and enhance growth across a range of plant species^17^. *Astragalus mongholicus* infected with root rot disease recovered as a result of colonization with a simplified bacterial community which triggered an induced disease resistance response in the plant^18^. A single bacterial maintained root growth in a complicated microbiome^5^. However, analyzing the interaction to understand how microbes affect plant phenotype is still in its infancy. If and how the interaction between bacteria and their host plant is modulated by secondary metabolites of the host plant is unclear. Many studies of the changes in the plant phenotype in response to different biotic factors have been reported^2,19, 20^. It has also been reported that microbial colonization can change the flowering time of *Arabidopsis thaliana*, whereas the root microflora contributes to plant nutrient uptake and resistance to biotic stresses, and tolerance to abiotic stresses^21,22^. Elimination of the floral microflora affected the composition and proportions of the terpenes in the volatiles^23^. The inoculation of endophytic bacteria *Bacillus aquimaris* and *Micrococcus luteus* into plants of Jerusalem artichoke (*Helianthus tuberosus*) increased height, stem and root weight, root length, root diameter, root volume, root area, and root surface area^24^, whereas colonization of maize seedlings by four strains of plant growth-promoting rhizobacteria significantly increased the root dry weight under abiotic stress conditions^25^.

Some reports have shown changes in the plant secondary metabolite in response to inoculation with PGPB. Secondary metabolite accumulation of grapevine treated with the bacterium *Enterobacter ludwigi*i showed a remarkable increase in the level of vanillic acid and a decrease in the concentration catechin, arbutin, esculin, astringin, ampellopsin, pallidol, isohopeaphenol, and D-quadrangularin. Changes in the levels of procyanidin 1, epicatechin, taxifolin and the sum of quercetin-3-galactoside and quercetin-3-glucoside in roots and stems were detected^26–28^. *Bacillus amyloliquefaciens* FZB42 is a gram-positive soil bacterium with the potential to produce non-ribosomal secondary metabolites; it has been suggested that 8.5% of the *B. amyloliquefaciens* FZB42 genome encodes enzymes for the non-ribosomal biosynthesis of secondary metabolites, emphasizing that these compounds are closely related to the lifestyle of this bacterium^29^. *Pseudomonas chlororaphis* PCL 1606 is a bacterium isolated from the rhizosphere of avocado trees^30^, and it can produce several low-molecular-weight defense molecules, including hydrogen cyanide, 2-hexyl-propyl-resorcinol, and pyrrolnitrins, resulting in antifungal and antimicrobial activities^31^. Meanwhile, *P. chlororaphis* has been reported to be capable of producing biocontrol-related siderophores such as achromobactin, pyochelin, and pyoverdine^32–34^. So far, bacteria-mediated changes in phytochemistry reported involve the model plant species *A. thaliana*, food crops, medicinal plants, trees/shrubs and ornamentals^26^. The use of plant growth-promoting bacteria (PGPB) as a biological fertilizer to improve plant growth and development, and to increase the concentrations of their health-related secondary metabolites in medicinal plants would be a novel strategy.

Plants are attacked by microorganisms as a form of biotic stress with disease-tolerant plants producing increased activities of antioxidant enzymes (e. g., peroxidase (POD) and superoxide dismutase (SOD)) to mitigate disease-related oxidative stress^35^. Malondialdehyde (MDA) is a strong indicator of cell damage caused by membrane lipid peroxidation under a variety of stress conditions, and its content can indirectly reflect the degree of peroxide damage^36^. To date, there is no conclusion as to whether and how PGPB might increase SOD and POD activities, and MDA content.

*Houttuynia cordata* Thunb. belongs to the family Saururaceae and is a dual-purpose plant, which can be used as both food and medicine, being particularly rich in pharmaceutically active secondary metabolites. It has been reported that *H. cordata* contains several secondary metabolites, including volatile terpenes and flavonoids, some of which exhibit pharmaceutical activities (e.g., antibacterial, antiviral, anti-inflammatory, immunological, anticancer, and antimutagenic effects)^37–39^. Microbial species (mostly bacteria, but including only three fungal species, namely *Ilyonectria liriodendri*, an unidentified fungal species and *Penicillium citrinum*) have been isolated and identified from healthy *H. cordata* tissues and re-inoculation with these fungal species promoted the growth and development of *H. cordata*^28^, However, the effects of endophytic bacteria in *H. cordata* have not been directly examined. If PGPB isolated from *H. cordata* can be shown to be a biological fertilizer, causing increasing concentrations of the pharmaceutically active secondary metabolites and promoting the growth of *H. cordata*, this may prove to be a novel route for mining herbal medicines from *H. cordata*.

In this study, we hypothesized that there are interactions between individual PGPB strains and *H. cordata* growth and development (e.g., phenotype) by bacterial induction of disease resistance and/or oxidative stress tolerance by upregulation of enzymic and non-enzymic antioxidants. Specifically, we hypothesized that: (1) *H. cordata* morphology changed in response to colonization by PGPB; (2) the concentrations of secondary metabolites changed in response to PGPB colonization, and (3) the MDA concentration and SOD and POD activities changed in response to PGPB colonization*.* The current study provided basic data to assess the linkages between endophytic bacteria, plant phenotype and metabolites of *H. cordata* to provide an insight into PGPB use as biological fertilizer, promoting the synthesis of medicinal plant compounds*.*

## Materials and methods

### Isolation and identification of the bacteria in *H. cordata* roots

The soil was removed from the roots of *H. cordata* (harvested from Kaiyang County, Guizhou Province, China; 26° 35′ N, 106° 42′ E), which were then rinsed with running water for 2 h, and then rinsed with deionized water three times. Following the published methodology^36^, the roots of *H. cordata* were cut into 0.3 cm long fragments, sterilized with 75% (v/v) ethanol for 30 s and 0.1% (w/v) mercuric chloride for 6 min, then rinsed with sterile water five times and dried with sterile filter paper. The sterilized roots were cut into pieces, and then extracted in 0.5 mmol/L phosphate-buffered saline (PBS) by ultrasonic vibration for 10 min. The root extract in PBS was diluted into 10^−5^, 10^−6^, and 10^−7^ concentrations. A 0.1 mL aliquot of each concentration was added and spread onto nutrient agar (NA, containing 5.0 g peptone, 18 g agar, 5.0 g NaCl, 1,000 mL distilled water, pH 7.0–7.2), with five replicate plates for each concentration. All NA plates were incubated at 28 °C for 3 days. This procedure was repeated until a pure strain was obtained. The purified bacteria were cultured on NA medium for 7 days at 28 °C for and identified by morphological characteristics, with gene sequencing used to identify the strain. The primers used were: 7F: CAGAGTTTGATCCTGGCT; 1540R: AGGAGGTGATCCAGCCGCA and, finally, the sequence was compared by a BLAST search in GenBank and compared with CLUSTAL X software by using the 1000 replicate the program. MEGA7 software was used to construct a phylogenetic tree by the Neighbor-Joining (NJ) method^40^.

### In-vitro culture of sterile H. cordata seedlings

In our study, all the seeds of *H. cordata* come from the same plant growing in Kaiyang County, Guizhou Province (26° 31′ 35″ N, 107° 14′ 36″ E). Seeds were sterilized with 75% (v/v) methanol for 30 s, sterilized with 0.2% (w/v) HgCl_2_ for 5 min, and then washed with sterile, deionized water five times. Then, the seeds were inoculated into a culture flask (containing ½ Murashige and Skoog (MS) containing 0.1 mg/L kinetin + 0.1 mg/L 1-naphthyl acetic acid + 0.1 mg/L gibberellic acid). An aliquot (0.2 mL) of the last rinse water was cultured on ½ MS culture medium as the control, to verify the effect of seed sterilization and ensure that the seedlings were sterile. The sterile seeds were cultured in an incubator for 45 days and then germinated under controlled conditions (light intensity of 1500–2000 lx light, 23 ± 2 °C, 12 h light/12 h dark cycle) to obtain the seedlings of the first generation. The second-generation seeds were obtained by inoculating the first-generation under the same conditions.

### Screening assay of the promoting *H. cordata* growth bacteria

To evaluate whether the bacteria isolated can promote the growth of *H. cordata*, its sterile seedlings were randomly divided into sterile culture bottles (½ MS culture medium) as either a control group or an experimental group. The bacterial number was calculated by the plate colony counting method and its concentration was adjusted to 2 × 10^6^ CFU/mL and then directly injected into the sterile culture bottle with an injection volume of 5 mL to represent the experimental group, respectively. The control group involved the injection of 5 mL sterile water under the same conditions, with three replicates of each of the experimental and the control groups. The seedlings were incubated in the plant growth room for 45 days. Finally, the *H. cordata* growth was observed and the morphometry was recorded, respectively.

### Inoculation of *H. cordata *with *P. fluorescens*

The method, which evaluate the effect of *P. fluorescens* inoculation on the *H. cordata* growth, was the same as in Screening assay of the promoting *H. cordata* growth bacteria. Finally, the growth of *H. cordata* was observed, and the number of lateral roots, plant height, fresh weight and dry weight were recorded.

### *P. fluorescens’s* IAA production assay

The capacity of *P. fluorescens* to produce IAA was determined by the colorimetric method using the Salkowski reagent. Briefly, *P. fluorescens* (OD_600_ = 0.2) was inoculated in the NA medium containing L-tryptophan (200 mg L^−1^), incubated for 3 d at 28 °C, and the OD_600_ value was determined. The bacterial suspension was centrifuged at 1000 r min^−1^ for 10 min and then 1 mL of the suspension was added to the colorimetric solution and incubated in the dark for 30 min. Absorbance of the suspension was measured at 530 nm. A standard solution of IAA (2‒10 mg L^−1^) was prepared in the same way, and sterile water was prepared in the same way (CK). a calibration curve was plotted to calculate IAA secreted by *P. fluorescens*.

### Determination of MDA concentration in *H. cordata*

The concentration of MDA in *H. cordata* seedlings was analyzed by a published method^28^. A sample of 200 mg leaf fresh weight was homogenized in 10 mL of 10% (w/v) trichloroacetic acid, followed by centrifugation at 3000 rpm for 10 min, after which 2 mL supernatant was added to 2 mL of 0.6% (w/v) thiobarbituric acid. The mixture was heated for 15 min to 100 °C, then quickly cooled on ice and centrifuged as before. The absorbances of the supernatant at 532, 600 and 450 nm were recorded, and the concentration of MDA (C_MDA_) in the extract was determined from the equation: C_MDA_ (μmol/L) = [6.45 × (A_532_ − A_600_) − [0.56 × A_450_)].

### Determination of antioxidant enzyme activities in *H. cordata*

SOD activity was quantified by the nitroblue tetrazolium method^28^. A sample (200 mg) of sliced fresh leaf tissue was ground with 0.05 mM phosphate-buffered saline (PBS) at pH 7.8, and the extract was centrifuged at 4,000 rpm for 10 min before a 50 μL aliquot of the supernatant was added to the reaction mixture containing 13 mM methionine, 75 μM NBT, 2 μM riboflavin and 10 μM EDTA Na_2_, adjusted to a 3 mL reaction volume. After exposing the reaction mixture to a fluorescent lamp for 20 min (4000 lx), the absorbance of the reaction mixture at 560 nm was measured to assay the SOD activity, in terms of the suppression ratio: Suppression ratio (%) = [(A0 − Ai)/(A0)] × 100, where A0 is the absorbance of the control reaction volume in the absence of the leaf extract, and Ai is the absorbance of the treatment reaction volume in the presence of the leaf extract. One unit (1 U) of SOD activity was described as that concentration that achieved 50% inhibition.

Determination of POD activity was carried out according to a published method^28^. Leaf (100 mg fresh weight) was ground with 5 mL 40 mM PBS at pH 6.0 and the homogenate was centrifuged at 4000 rpm for 15 min. A 50 μL aliquot of the supernatant was added to a reaction mixture consisting of 2 μM H_2_O_2_ and 9 mM guaiacol, adjusted to a total volume of 5 mL with PBS, pH 6.0. Changes in absorbance at 470 nm were recorded every 30 s for 3 min. POD enzyme activity was presented in terms of activity units, where one unit of the enzyme (1 U) was represented by an increase in A_470_ of 0.01 per min. Three biological replicates were measured for each treatment, each replicate representing one seedling.

### Assay of anti-*P. fluorescens* activity of *H. cordata* extracts

The purified *P. fluorescens* were suspended in a centrifuge tube filled with 9 mL of sterile water by an inoculation ring and then diluted to a 1 × 10^6^ CFU/mL suspension. A sample of 20 g of fresh *H. cordata* seedling tissue was weighed and added into a 100 mL beaker, followed by 100 mL of methanol, which was sealed with film and placed in an ultrasonic instrument for 30 min at 25 °C. The *H. cordata* extract obtained was filtered, concentrated by rotary evaporation for 10 min, and then stored in a sterile test tube under sterile conditions at 4 °C. The inhibitory effect of *H. cordata* extracts on *P. fluorescens* was determined by the Oxford cup assay. In short, 0.1 mL of bacterial suspension liquid was suspended in a beef paste peptone culture medium under sterile conditions and cultured by a coating plate method, with an Oxford cup vertically arranged on the culture medium. Next, 0.2 mL of the *H. cordata* extract was added to the Oxford cup, which was then placed in a bacterial incubator for 48 h culture. Two groups of controls were set up: one group was inoculated with *P. fluorescens* only, and the other group was inoculated with sterile water only. Finally, the diameter of the bacteriostatic ring of the different media was measured with vernier calipers.

### Analysis of phenolics in *H. cordata* leaves

Fresh *H. cordata* leaves (≈ 0.1 g) were placed in a brown bottle, where 2 mL 70% (v/v) methanol was added, exposed to ultrasonic extraction for 30 min, and the extracted sample was filtered through a 0.2 µm nylon membrane filter. The concentrations of chlorogenic acid, afzelin, rutin, isoquercitrin and quercetin in fresh *H. cordata* leaves were determined using High-Performance Liquid Chromatography (HPLC) LC-20AT (Shimadzu, Japan). Stock solutions (0.01%) of markers chlorogenic acid, afzelin, rutin, isoquercitrin, quercitrin and quercetin (purchased from J&K Scientific) were prepared in ethanol and stored at 4 °C. The analysis conditions were as follows. The chromatographic column was reversed-phase Shim-pack CLC-ODS (150 mm × 6.0 mm × 5 μm, No. 61627330). The linear gradient elution consisted of eluent A (methanol: acetonitrile = 5:11 (v/v)) and eluent solvent B (0.1% methane acid (v/v)): 13% A (0–6 min); 17% A (6–9 min); 19% A (9–11 min); 29% A (1–31 min); 38% A (31–48 min); 100%A (48–54 min); 13% A (54–75 min). The flow rate program was 1.40 mL/min (0–6 min); 1.40–0.62 mL/min (6–11 min); 0.62–0.82 mL/min (11–48 min); 0.62–1.40 mL/min (48–51 min); and 1.4 mL/min (51–75 min). The detection wavelength was 345 nm and the column temperature was 35 °C. Working solutions of the samples were obtained by further diluting the sample solutions and standard stock solutions with the appropriate concentration of methanol. The working solutions were analyzed by HPLC. The chlorogenic acid, afzelin, rutin, isoquercitrin, quercitrin and quercetin contents in the samples were calculated based on the peak areas.

### Analysis of major volatiles in *H. cordata*

Sample extraction involved placing 0.1 g of fresh *H. cordata* leaves in a brown bottle, to which 2 mL of 70% (v/v) dichloromethane (CH_2_Cl_2_) were added, followed by ultrasonic extraction for 30 min and filtration, with naphthalene included as the internal standard. All extracts were stored at 4 °C until assayed. The stock solutions (1 mg/mL) of the volatile markers (*α*-pinene, camphene, *β*-pinene, *β*-myrcene, limonene, eucalyptol, *trans*-2-hexenal, *cis*-3-hexenyl-acetate, hexanol, *cis*-3-Hexen-1-ol, *trans*-2-Hexen-1-ol, decanal, linalool, bornyl acetate, *β*-caryophyllene, 2-undecanone, borneol, decanal and 3-tetradecanone) were prepared with an appropriate methanol concentration. Concentrations of *H. cordata* volatiles were determined by gas chromatography-mass spectrometry (GC–MS; GCMS-QP2010, Shimadzu, Japan). Capillary columns (Factor Four TM: VF-WAXms, 30 m × 0.25 mm × 0.25 µm). The GC conditions were as follows: injector temperature: 250 °C; flow rate: 1.11 mL/min; injection method: not split. The column temperature was 35 °C (3 min), 5.6 °C/min to 100 °C (1 min), 2.65 °C/min to 125 °C, 15 °C/min to 230 °C (5 min). The MS conditions were as follows: ion source: EI; ionization voltage: 70 eV; ion source temperature: 210 °C; interface temperature: 250 °C; scan mode: SIM; solvent delay time: 5 min. To allow calculation of the correction factor, naphthalene was added to both standard solutions and to sample extracts in equal amounts as an internal standard. The concentration of each volatile was calculated by comparing the peak area of the quantifier ions. Three biological replicate samples were measured for each treatment.

### Statistical analysis

Microsoft Office Excel 2010 was used to analyze the raw data, with each parameter being measured in three independent biological replicates per sample. Summary statistics were presented as mean ± standard deviation. Data analysis was carried out by one-way analysis of variance (ANOVA), followed by Tukey test to identify significant differences between individual samples, using the R language.

## Results

### Identification of endophytic bacteria isolated from *H. cordata* roots

**Figure 1:**
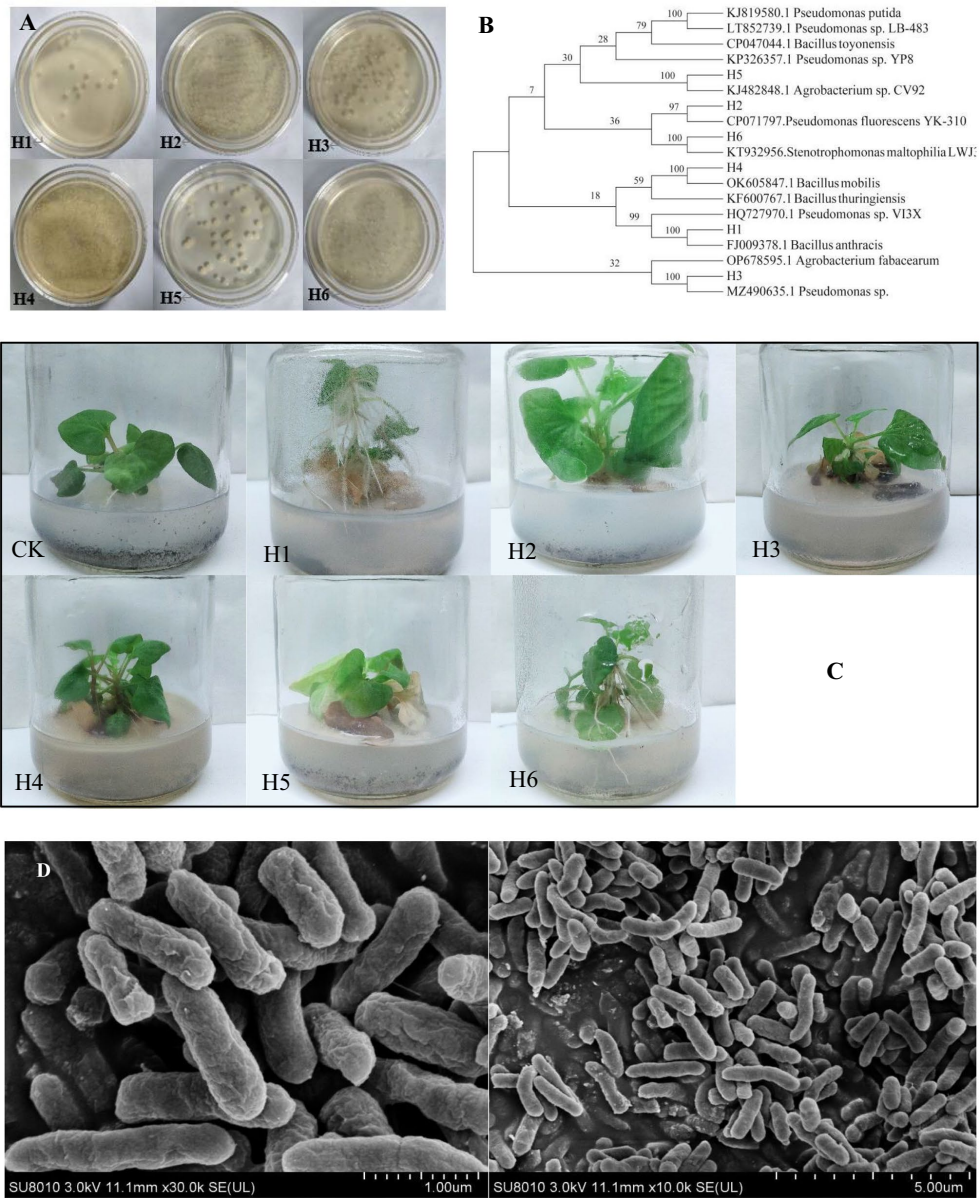
(**A**) 6 candidates composing bacteria isolated from *H. cordata*; (**B**) Phylogenetic trees showing the relationships of strains to closer species of *H. cordata* based on analysis of the 16S rRNA gene sequences of the bacteria and 6 candidates composing bacteria were identified as H1 = FJ009378.1 *Bacillus anthracis*; H2 = CP071797. *Pseudomonas fluorescens* YK-310; H3 = Mz490635.1 *Pseudomonas* sp.; H4 = OK605847.1 *Bacillus mobilis*; H5 = HJ482848.1 *Agrobacterium* sp. CV92; H6 = KT932956.*Stenotrophomonas maltophilia* LWJ3. (**C**) The effects of 6 candidates composing bacteria on *H. cordata*. Notes: CK, H1: inoculation with *B. anthracis*; H2: inoculation with *P. fluorescens*; H3: inoculation with *Pseudomonas* sp.; H4: inoculation with *B. mobilis*; H5: inoculation with *Agrobacterium* sp.; H6: inoculation with *S. maltophilia*. (**D**) The morphology of the bacterium strain *P. fluorescens* (H2).

Six different bacterial strain (named H1–H6) were isolated and purified from the roots of *H. cordata* plants (Fig. 1A). BLAST search results of the bacterial strain genome sequences showed that the DNA sequence of these bacterial strains were more than 97% identical to that of FJ009378.1 *Bacillus anthracis* (H1), CP071797. *Pseudomonas fluorescens* YK-310 (H2), Mz490635.1 *Pseudomonas* sp. (H3), OK605847.1 *Bacillus mobilis* (H4), HJ482848.1 *Agrobacterium* sp. CV92 (H5) and KT932956.*Stenotrophomonas maltophilia* LWJ3 (H6) (Fig. 1B,C). Six bacterial strains were tentatively identified as *Bacillus anthracis* (H1), *Pseudomonas fluorescens* (H2), *Pseudomonas* sp. (H3), *Bacillus mobilis* (H4), *Agrobacterium* sp. (H5) and *Stenotrophomonas maltophilia* (H6), respectively.

### Screening of the promoting bacteria

According to the results of the potential promotion experiments, *P. fluorescens* was preliminarily verified to promote the growth of *H. cordata* seedling (Fig. 1C). Therefore, *P. fluorescens* (Fig. 1D) was used as a PGPB of *H. cordata* in the following experiments.

### IAA of production of *P. fluorescens*

The results showed that the test tubes had a light pink color in Salkowski's color reaction, and *P. fluorescens* could secrete IAA (Supplementary Fig. S1).

### Morphological effects on *H. cordata *seedlings inoculated with *P. fluorescens*

**Figure 2:**
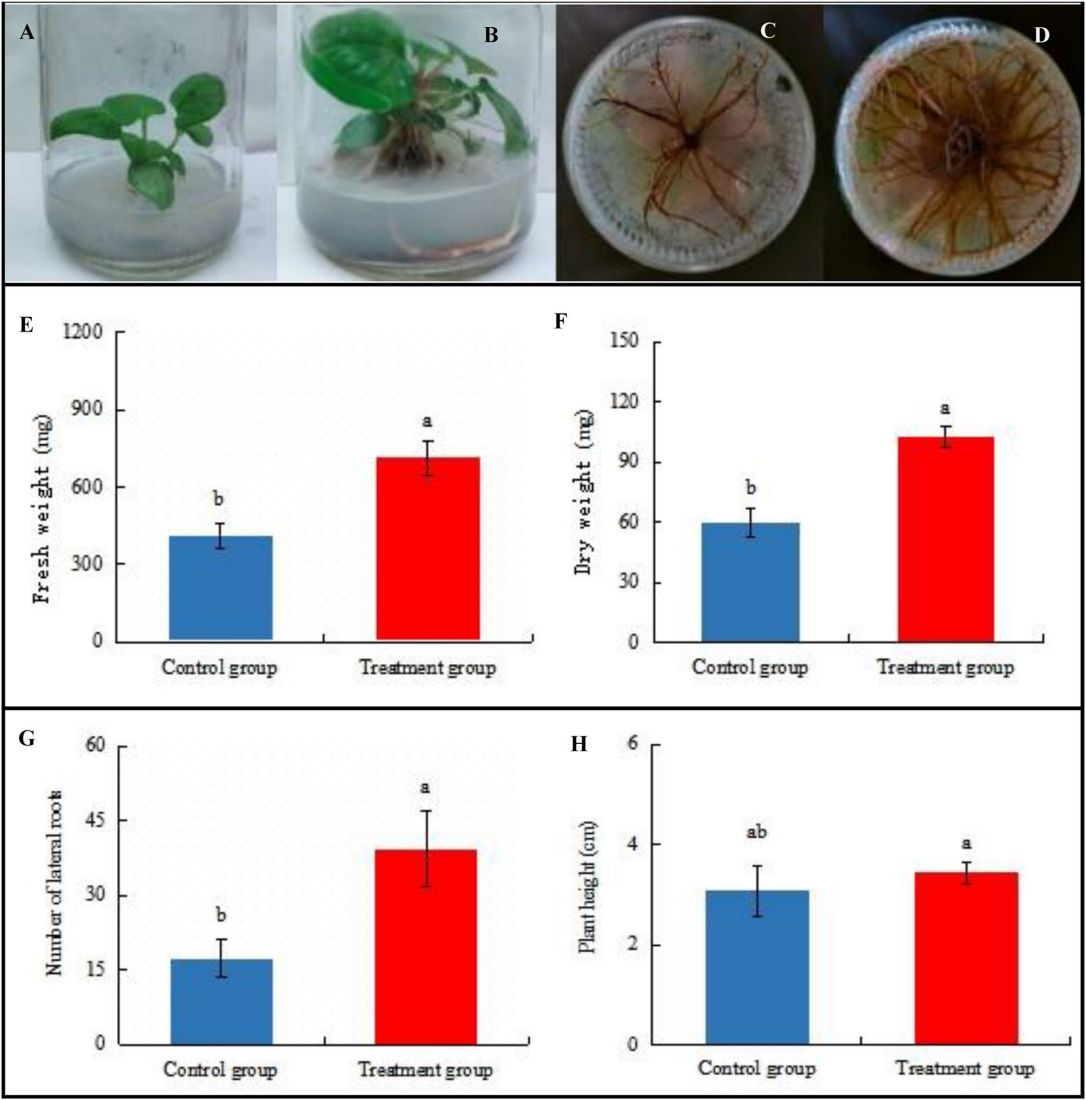
Effect of inoculating *P. fluorescens* on physiological morphology of *H. cordata*. (**A**) The control group: *P. fluorescens*-non-inoculated *H. cordata* seedling cultured after 45 days, (**B**) Treatment group: *H. cordata* seedlings inoculated with *P. fluorescens* and cultured after 45 days, (**C**) Control group: *H. cordata* seedlings roots that non-inoculated *P. fluorescens* and cultured after 45 days, (**D**) Treatment group: *H. cordata* seedlings roots which inoculated with *P. fluorescens* and cultured after 45 days. The results are presented as mean SD, the error bar represents the standard deviation of biological parallelism in the figure. (*n* = 3, *P* < 0.05).

Inoculation with *P. fluorescens* for 45 days resulted in the morphological effects on *H. cordata* seedlings, relative to the uninoculated control seedlings. The morphology of *H. cordata* seedlings inoculated with the *P. fluorescens* strain was markedly superior to that of the control (Fig. 2A,B), The leaf blade was wider and larger, and the color of the leaves was darker green. In addition, the roots and stems were also significantly thicker, the number of lateral roots was higher, the root surface area was greater, the number of lateral roots was much higher than that of the control group (Fig. 2C,D), and the fresh weight and dry weight of the roots of samples treated with *P. fluorescens* were significantly higher than those of the control. The total fresh weight per seedling between the control seedlings and *P. fluorescens*-inoculated seedlings treatments ranged from 409.3 to 711.9 mg (higher 174%) (Fig. 2E), the total seedling dry weight ranged from 59.4 to 102.5 mg (higher 172%) (Fig. 2F), and the number of lateral roots ranged from 17.3 to 39.3 cm (higher 227%) (Fig. 2G). In contrast, the plant height of *H. cordata* was not significantly different from that of the control (Fig. 2H).

### Physiological effects on *H. cordata* seedlings inoculated with* P. fluorescens*

**Figure 3:**
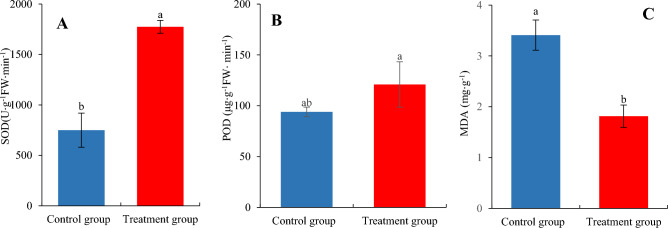
Effects on physiological of *H. cordata* inoculated with *P. fluorescens*. (Control: *H. cordata* seedlings that non-inoculated *P. fluorescens* and cultured after 45 days; (*P. fluorescens*: *H. cordata* seedlings inoculated with *P. fluorescens* and cultured after 45 days). The value represents means ± standard deviation (*n* = 3). Any two samples in the same bar chart with a different lowercase letter on the bar have significantly different mean values (*P* < 0.05).

Lipid peroxidation is a consequence of oxidative stress, and the MDA concentration of *H. cordata* seedlings inoculated with *P. fluorescens* was 53% lower than that in control seedlings, reaching 1.6 mg g^−1^ (*P* < 0.05, Fig. 3A). The activity of SOD (1774.7 U g^−1^ FW h^−1^) in the *P. fluorescens*-inoculated *H. cordata* seedlings was 137% higher than that in control seedlings (749.8 U g^−1^ FW h^−1^) (*P* < 0.05, Fig. 3B), while the activity of POD (120.9 U g^−1^ min^−1^) was also significantly (29%) higher in *P. fluorescens*-inoculated *H. cordata* seedlings by 29% than in control seedlings (93.9 U g^−1^ min^−1^) (*P* < 0.05, Fig. 3C).

### Anti-bacterial activities of *H. cordata* extract toward *P. fluorescens*

**Figure 4:**
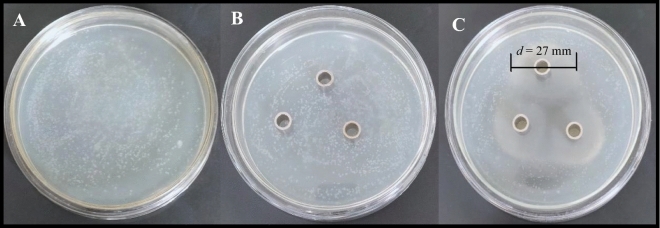
Assay of *H. cordata* extracts to anti-bacterial activities. (Three groups of controls were set, A group was only inoculated with *P. fluorescens* only, B group was inoculated with *P. fluorescens* and 0.2 ml sterile distilled water in the Oxford cup, C group that 0.2 mL of *H. cordata* extract taken in the oxford cup and then placed in a bacterial incubator for culture for 48 h. Finally, the diameter of the bacteriostatic ring of the extract was measured with a vernier caliper).

We investigated the interactions between *P. fluorescens* and the extracts of *P. fluorescens*-inoculated *H. cordata* extracts in terms of the anti-bacterial activities of the extracts toward *P. fluorescens *in vitro (Fig. 4A–C). The bacteriostatic ring diameter of *H. cordata* extracts against *P. fluorescens* was 27 mm (Fig. 4C).

### Effects on the concentrations of phenolics in *H. cordata *seedlings inoculated with *P. fluorescens*

**Figure 5:**
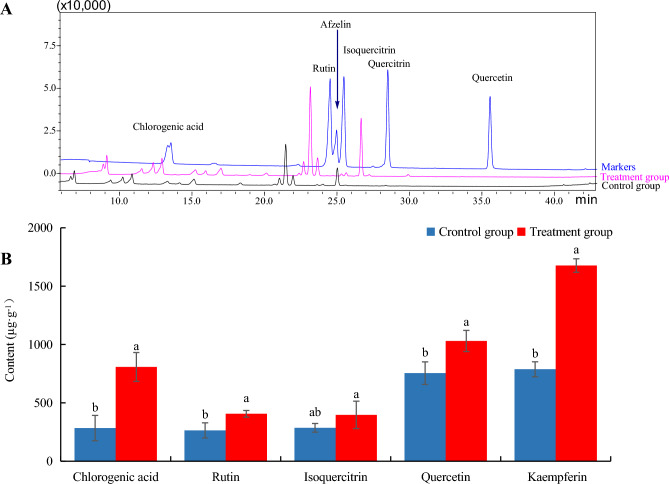
(**A**) HPLC chromatographic profiles of *H. cordata* phenolics (markers represent standard compounds of phenolics; *P. fluorescens* represent *H. cordata* seedlings inoculated with *P. fluorescens* and cultured after 45 days; The control represents *H. cordata* seedlings which were not inoculated with *P. fluorescens* and cultured for 45 days). (**B**) Effects of *P. fluorescens* on phenolics in *H. cordata* seedlings (*P. fluorescens*: *H. cordata* seedlings which inoculated with *P. fluorescens* and cultured after 45 days; Control: *H. cordata* seedlings that were not inoculated with *P. fluorescens* and cultured for 45 days). The results are presented as mean SD, the error bar represents the standard deviation of biological parallelism in the figure. (*n* = 3, *P* < 0.05).

The effect on the concentrations of the major phenolics (including chlorogenic acid, afzelin, rutin, isoquercitrin, quercitrin and quercetin) in *H. cordata* seedlings in response to inoculation with *P. fluorescens* was studied. In seedlings inoculated with *P. fluorescens*, the concentrations of chlorogenic acid (807.5 μg/g), rutin (406.3 μg/g), quercitrin (1030.3 μg/g) and afzelin (1676.5 μg/g) were 284%, 154%, 137% and 213% higher than in control seedlings, which exhibited concentrations of 284.0, 264.2, 754.5 and 787.7 μg/g, respectively (*P* < 0.05), but the effect on the concentration of isoquercitrin was not significant compared with that of the control, non-colonized seedlings (*P* > 0.05) (Fig. 5A,B.

### Effects on the concentrations of the volatiles in *H. cordata* seedlings inoculated with *P. fluorescens*

**Figure 6:**
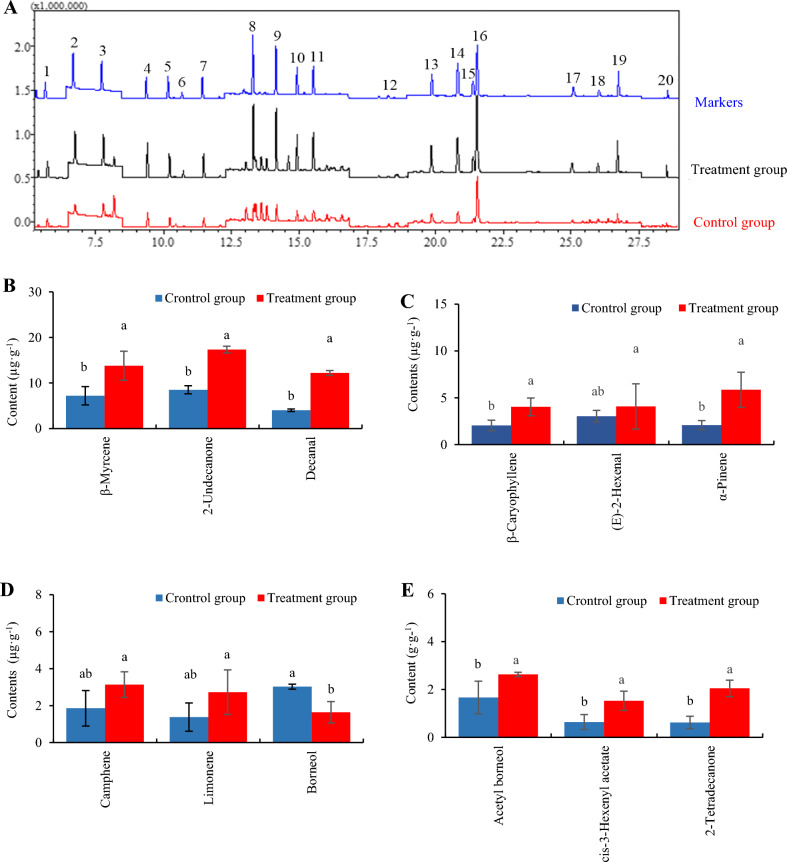
(**A**) Gas chromatographic-mass spectrogram (GC–MS) profiles of *H. cordata* volatiles (standard represent standard compounds of volatiles; *P. fluorescens* represent *H. cordata* seedlings inoculated with *P. fluorescens* and cultured after 45 days; control represent *H. cordata* seedlings which were not inoculated with *P. fluorescens* and cultured for 45 days). (1, α-Pinene; 2, Camphene; 3, β-Pinene; 4, β-Myrcene; 5, Limonene; 6, Eucalyptol; 7, *trans*-2-Hexenal; 8, *cis*-3-Hexenyl-acetate; 9, Hexanol; 10, *cis*-3-Hexen-1-ol; 11, *trans*-2- Hexen-1-ol; 12, Decanol; 13, Linalool; 14, Bornyl acetate; 15, β-Caryophyllene; 16, 2-Undecanone; 17, Borneol; 18, Naphthalene; 19, Decanal; 20, 3-Tetradecanone). (**B**–**E**) Effects of bacteria *P. fluorescens* on volatile secondary metabolites in *H. cordata* seedlings (control: *P. fluorescens*-non-inoculated *H. cordata* seedlings cultured after 45 days; *P. fluorescens*: *H. cordata* seedlings inoculated with *P. fluorescens* and cultured after 45 days). The results are presented as mean SD, the error bar represents the standard deviation of biological parallelism in the figure. (*n* = 3, *P* < 0.05).

The 19 volatiles (*α*-pinene, camphene, *β*-pinene, *β*-myrcene, limonene, eucalyptol, *trans*-2-hexenal, hexanol, *cis*-3-hexenyl-acetate, *cis*-3-hexen-1-ol, *trans*-2- hexen-1-ol, decanal, linalool, bornyl acetate, *β*-caryophyllene, 2-undecanone, borneol, decanal and 3-tetradecanone) in *H. cordata* were analyzed, and *α*-pinene, camphene, *β*-myrcene, limonene, *trans*-2-hexenal, *cis*-3-hexenyl-acetate, bornyl acetate, *β*-caryophyllene, 2-undecanone, borneol, decanal and 3-tetradecanone were detected, with others not being detected. *P. fluorescens* had a significant stimulatory effect on the concentration of volatiles compared with the control, except for *trans*-2-Hexenal, camphene and limonene (Fig. 6C,E), with *β*-myrcene increasing by 192% (treatment: 13.77 μg/g, control: 7.19 μg/g), 2-undecanone by 203% (treatment: 17.33 μg/g, control: 8.51 μg/g), decanal by 304% (treatment: 12.19 μg/g, control: 4.01 μg/g), *β*-caryophyllene by 197% (treatment: 4.03 μg/g, control: 2.04 μg/g), *α*-pinene by 281% (treatment: 5.86 μg/g, control: 2.08 μg/g), bornyl acetate by 157% (treatment: 2.63 μg/g, control: 1.66 μg/g), *cis*-3-Hexenyl-acetate by 239% (treatment: 1.53 μg/g, control: 0.63 μg/g) and 3-tetradecanone by 328% (treatment: 2.05 μg/g, control: 0.62 μg/g) in *P. fluorescens-*inoculated *H. cordata* seedlings (Fig. 6B–D), compared with control seedlings (*P* < 0.05), but the concentration of borneol (1.64 μg/g) in *P. fluorescens-*inoculated *H. cordata* seedlings was 54% lower than in the control (3.03 μg/g) (*P* < 0.05, Fig. 6E).

### Experimental materials

The experimental research of plants (either cultivated or wild), including the collection of plant material, all comply with relevant institutional, national, and international guidelines and legislation.Results

## Discussion

Medicinal plants are a widely used resource throughout the world, especially in Chinese traditional medicine^41,42^. A major aim of researchers is to improve the yield of pharmacodynamic active compounds from a medicinal plant because of the potentially enormous medicinal and economic benefits^43^. However, most researchers focus on the effects of abiotic factors (planting, fertilizers, temperature, soil, etc.) on the concentrations of the bioactive compounds^44^, with biological factors (e.g., endophytic bacteria) usually being ignored. In this study, we isolated and identified a strain of the bacterium *P. fluorescens* (Fig. 1B and Supplementary Table S1), which had colonized rhizomes of wild *H. cordata.* However, before the current study, the effect of *P. fluorescens* on the phenotype and functional (phytochemical) traits of *H. cordata*had not been investigated*.*

Endophytic bacteria can improve the phenotype of the plants they colonize (e.g., variables of leaves, roots, stems, etc.)^5,16^. We found that the phenotype of *P. fluorescens*-inoculated *H. cordata* seedlings was markedly changed (with the leaf blade looking wider, larger and darker green) (Fig. 2A,B), with the roots and rhizomes also being significantly thicker, and the lateral root number and the root surface area increasing (Fig. 1C,D) due to *P. fluorescens* can secrete IAA (Supplementary Fig. S1) to promote *H. cordata* root numbers. The number of lateral roots and the total seedling fresh and dry weights were significantly increased (*P* < 0.05, Fig. 2E–G), suggesting that this *P. fluorescens* strain is a PGPB, an effect which may relate to the defensive and regulatory response to metabolite signaling. Such PGPB-induced plant growth-promoting activity is associated with the gibberellin-producing ability of PGPB^25^.

Bacteria can improve functional traits (e.g., MDA concentration, SOD, POD activities, and secondary metabolite concentrations) of plants^2,24^. Plants respond to the stresses of multiple biological factors (e.g., bacteria, fungi, and insect pests) by operating a defensive system; part of this involves antioxidant systems to protect plants and subsequent oxidative stress damage (e.g., SOD, POD, and non-enzymatic antioxidants)^12,45^. In the current study, we noted that, compared with control seedlings, the activities of SOD and POD in *P. fluorescens*-inoculated *H. cordata* seedlings were significantly increased (*P* < 0.05, Fig. 3A,B), indicating that *P. fluorescens* induces *H. cordata* to promote SOD and POD activities. Compared with the control seedlings, the MDA concentrations in *P. fluorescens*-inoculated seedlings were significantly decreased (*P* < 0.05, Fig. 3C), indicating that *P. fluorescens* can induce antioxidant effects to decrease MDA concentrations, reflected in reduced damage of membrane lipid peroxidation to the biological membrane structure and function, mainly the cell plasma membrane, thus preventing decreases in the membrane permeability, an effect which would be associated with good growth of *H. cordata*.

Plant secondary metabolites can directly prevent attack by bacteria or can modulate plant microbiota to achieve a similar effect with phenolics and volatiles being among the important bioactive compounds^46,47^. When *H. cordata* seedlings were inoculated with *P. fluorescens*, enhanced activities of the antioxidant enzymes, POD and SOD were observed, preventing oxidative stress damage. Meanwhile. The concentrations of several phenolics (chlorogenic acid, rutin, quercitrin and afzelin) in *H. cordata* seedlings inoculated with *P. fluorescens* were significantly increased, respectively (*P* < 0.05, Fig. 5A,B). The concentrations of the volatiles *β*-myrcene, 2-undecanone, decanol, *β*-caryophyllene, 3-tetradecanone, *α*-pinene, bornyl acetate, and γ-terpinene in *H. cordata* seedlings inoculated with *P. fluorescens* were significantly increased (*P* < 0.05, Fig. 6A–E), compared with control seedlings. These increases in non-enzymatic antioxidants further support the observation of reduced oxidative stress experienced by colonized *H. cordata*.

It has been reported that the concentrations of some antimicrobial plant secondary metabolites increase to mitigate biotic stress and that these compounds might inhibit pathogenic microbes in plants^26,47, 48^. We hypothesized that, if extracts of PGPB-colonized *H. cordata* could inhibit *P. fluorescens*, some antibacterial metabolites, the concentration of which increased in response to endophyte colonization, may play an important role in modulating *P. fluorescens* growth. In the experiment, there were significant dosage-dependent antibacterial activities in extracts of colonized *H. cordata* on *P. fluorescens* (Fig. 4A–C). Because the phenolics and volatiles in *H. cordata* were the dominant secondary metabolites and increased in concentration in extracts of colonized seedlings (Figs. 5A,B, 6B–E), this finding, that the extracts contained a large number of inducible phenolics and volatiles, which might inhibit *P. fluorescens*, suggests that PGPB colonization may contribute to biotic stress tolerance, which may contribute to the remarkably increased growth of *P. fluorescens*-inoculated *H. cordata* seedlings (Fig. 2A–D). These findings might suggest that there is a trade-off between the concentrations of *H. cordata* secondary metabolites and the demands for endophytic *P. fluorescens* growth within the seedlings to achieve growth, which may relate to signaling of the defense responses, so that biomass of *P. fluorescens* did not increase linearly due to the maintenance of a mutually beneficial balance between *H. cordata* and *P. fluorescens.*

The phenolics and volatiles analyzed in the current study are among the most pharmacodynamically active metabolites in *H. cordata*, exhibiting anti-inflammatory, antiviral, antibacterial and anticancer activities^49^. It has been reported that many endophytic and rhizosphere bacteria promote the growth of the host plant and its accumulation of secondary metabolites, revealing the potential value of beneficial bacteria in the productivity of medicinal plants^28,50–52^. Previous studies on the endophytic bacteria from *H. cordata* focused on the secondary metabolites and their antimicrobial activities^38,53^, whereas studies on the potential value of *P. fluorescens* as a PGPB and a biofertilizer, to stimulate plant growth, are lacking. In addition, the causal relationships between plant metabolome changes, plant growth promotion and/or antibacterial protection conferred by these PGPBs need further research.

## Conclusion

The findings from the current study showed that *P. fluorescens*, which was isolated from *H. cordata*, acts as a PGPB to promote *H. cordata* growth. Our results provide fundamental evidence to address the key questions regarding the association between bacteria and secondary metabolites in *H. cordata* and offer insight into their interaction. More importantly, these findings suggest that such beneficial microorganisms could be exploited to form a new generation of biological fertilizers, which could promote the synthesis of many medicinal plant compounds and stimulate the growth of the medicinal plants themselves.

### Supplementary Information

Supplementary Information.

## Data Availability

All data that support the findings of this study are available from the corresponding author, Yang Z. (email: yangzhannan@163.com), upon reasonable request.
