# Supplementary material for: Endophytic Pseudomonas fluorescens promotes changes in the phenotype and secondary metabolite profile of Houttuynia cordata Thunb
Source: Sci Rep. 2024 Jan 19;14:1710. doi: 10.1038/s41598-024-52070-y (PMC10798976; doi:10.1038/s41598-024-52070-y)
Supplement: Supplementary file 1 — Supplementary Information. [file 41598_2024_52070_MOESM1_ESM.docx]

**Supplementary Table and Fig. S**

**Supplementary Table S1** Genomic sequence of *P. fluorescens* isolated from *H. cordata*

| GenBank (accession) | Genome size | Genomic sequence |
| --- | --- | --- |
| CP071797 | 1429bp | ATGGCTCAGATTGAACGCTGGCGGCAGGCCTAACACATGCAAGTCGAGCGGATGAAGGGAGCTTGCTCCTGGATTCAGCGGCGGACGGGTGAGTAATGCCTAGGAATCTGCCTGGTAGTGGGGGACAACGTTTCGAAAGGAACGCTAATACCGCATACGTCCTACGGGAGAAAGCAGGGGACCTTCGGGCCTTGCGCTATCAGATGAGCCTAGGTCGGATTAGCTAGTTGGTGAGGTAATGGCTCACCAAGGCGACGATCCGTAACTGGTCTGAGAGGATGATCAGTCACACTGGAACTGAGACACGGTCCAGACTCCTACGGGAGGCAGCAGTGGGGAATATTGGACAATGGGCGAAAGCCTGATCCAGCCATGCCGCGTGTGTGAAGAAGGTCTTCGGATTGTAAAGCACTTTAAGTTGGGAGGAAGGGCAGTAAATTAATACTTTGCTGTTTTGACGTTACCGACAGAATAAGCACCGGCTAACTCTGTGCCAGCAGCCGCGGTAATACAGAGGGTGCAAGCGTTAATCGGAATTACTGGGCGTAAAGCGCGCGTAGGTGGTTCGTTAAGTTGGATGTGAAATCCCCGGGCTCAACCTGGGAACTGCATCCAAAACTGGCGAGCTAGAGTATGGTAGAGGGTGGTGGAATTTCCTGTGTAGCGGTGAAATGCGTAGATATAGGAAGGAACACCAGTGGCGAAGGCGACCACCTGGACTGATACTGACACTGAGGTGCGAAAGCGTGGGGAGCAAACAGGATTAGATACCCTGGTAGTCCACGCCGTAAACGATGTCAACTAGCCGTTGGGAGCCTTGAGCTCTTAGTGGCGCAGCTAACGCATTAAGTTGACCGCCTGGGGAGTACGGCCGCAAGGTTAAAACTCAAATGAATTGACGGGGGCCCGCACAAGCGGTGGAGCATGTGGTTTAATTCGAAGCAACGCGAAGAACCTTACCAGGCCTTGACATCCAATGAACTTTCCAGAGATGGATTGGTGCCTTCGGGAACATTGAGACAGGTGCTGCATGGCTGTCGTCAGCTCGTGTCGTGAGATGTTGGGTTAAGTCCCGTAACGAGCGCAACCCTTGTCCTTAGTTACCAGCACGTTATGGTGGGCACTCTAAGGAGACTGCCGGTGACAAACCGGAGGAAGGTGGGGATGACGTCAAGTCATCATGGCCCTTACGGCCTGGGCTACACACGTGCTACAATGGTCGGTACAAAGGGTTGCCAAGCCGCGAGGTGGAGCTAATCCCATAAAACCGATCGTAGTCCGGATCGCAGTCTGCAACTCGACTGCGTGAAGTCGGAATCGCTAGTAATCGCGAATCAGAATGTCGCGGTGAATACGTTCCCGGGCCTTGTACACACCGCCCGTCACACCATGGGAGTGGGTTGCACCAGAAGTAGCTAGTCTAACCTTC |


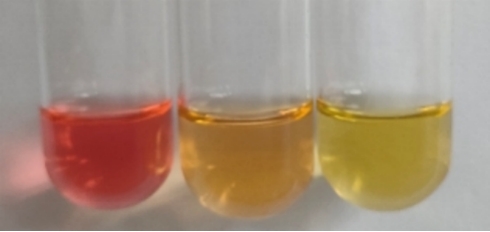


C

**CK**

***P. fluorescens***

**IAA**

B

A

**Supplementary Fig. S1.** Assay of the producing IAA capacity of *P. fluorescens*. **B.** *P. fluorescens* (OD_600_=0.2) was inoculated into the NA liquid medium containing L-tryptophan (200 mg·L^-1^), and incubated for 3 d at 28 ℃, the OD_600_ value was determined, and the suspension was centrifuged at 1000r/min for 10 min, and 1mL was added into the colorimetric solution and darkened for 30 min. After centrifugation of the suspension at 2000 r·min^-1^ for 10 min, 1mL of the suspension was added into the colorimetric solution for dark treatment for 30 min, and the absorbance value was measured at 530 nm. **A.** 2‒10 mg·L^-1^ IAA standard solution was prepared in the same way. **C.** Sterile water was prepared in the same way (CK). Salkowski's color reaction showed that the test tubes had a light pink color. *P. fluorescens* could secrete IAA.
